# Association between adjunctive use of an absorbable polymer tissue sealing membrane and postoperative subcutaneous fluid collection after posterior fossa surgery

**DOI:** 10.1097/MD.0000000000050621

**Published:** 2026-09-25

**Authors:** Weilong Chen, Zhenghao Liu, Xin Mei, Jina Chen, Yuefei Deng

**Affiliations:** a Department of Neurosurgery, Puning People’s Hospital, Jieyang, Guangdong, 515300, China; b Department of Neurosurgery, Sun Yat-Sen Memorial Hospital, Sun Yat-Sen University, Guangzhou, Guangdong, China.

**Keywords:** dural closure, posterior fossa surgery, retrospective cohort, subcutaneous fluid collection, tissue sealing membrane, wound complications

## Abstract

Postoperative subcutaneous fluid collection is an important wound-related complication after posterior fossa surgery and may delay recovery, necessitate additional interventions, and prolong hospitalization. Strategies that improve dural closure integrity may reduce postoperative fluid accumulation. This retrospective cohort study included 237 adult patients who underwent posterior fossa craniotomy at Sun Yat-sen Memorial Hospital, Sun Yat-Sen University, between January 2023 and January 2025. Patients were classified according to the dural closure technique used in routine clinical practice: conventional dural suturing plus an absorbable polymer tissue sealing membrane (n = 80) or conventional dural suturing alone (n = 157). Membrane use was not randomized, and a standardized indication for its use was not documented in the retrospective dataset. The primary outcome was a clinically diagnosed postoperative subcutaneous fluid collection within 3 months after surgery. No uniform biochemical or imaging criterion was prespecified to establish the cerebrospinal fluid (CSF) origin of every collection; therefore, the endpoint was not defined as confirmed CSF leakage or pseudomeningocele. Secondary outcomes included hospitalization duration, total surgical costs, and epithelialization time. Logistic regression was performed to identify factors associated with postoperative subcutaneous fluid collection. Postoperative subcutaneous fluid collection occurred in 6 of 80 patients (7.5%) in the observation group and 29 of 157 patients (18.5%) in the control group (*P* = .024). The observation group had a shorter postoperative hospital stay than the control group (8.2 ± 2.1 vs 10.5 ± 3.4 days, *P* = .012). No significant between-group differences were observed in total surgical costs or epithelialization time. Multivariable logistic regression showed that use of the sealing membrane was associated with lower odds of postoperative subcutaneous fluid collection, whereas older age and paramedian incision were associated with higher odds. Adjunctive use of an absorbable polymer tissue sealing membrane during dural closure was associated with a lower incidence of postoperative subcutaneous fluid collection and a shorter hospital stay after posterior fossa surgery, without a significant increase in surgical costs. Because treatment allocation was non-randomized and the fluid collections were not uniformly confirmed as CSF by a standardized biochemical or imaging criterion, these findings should be interpreted as an association and require prospective validation.

## 1. Introduction

Posterior fossa surgery is widely used in the treatment of intracranial tumors, vascular malformations, and disorders associated with increased intracranial pressure.^[1,2]^ Although advances in microsurgical techniques and perioperative management have markedly improved surgical safety, postoperative complications continue to represent an important challenge affecting patient recovery and prognosis. Among these complications, postoperative subcutaneous fluid collection is relatively common and has been reported to adversely influence postoperative wound healing and overall clinical outcomes.^[3,4]^ Clinically, subcutaneous fluid collection may result in local wound tension, pain, delayed incision healing, and an increased risk of infection. In more severe cases, repeated puncture, prolonged drainage, or secondary surgical intervention may be required, leading to prolonged hospitalization and increased medical costs.^[3,4]^

The development of subcutaneous fluid collection following posterior fossa surgery is generally considered to be multifactorial. Previous studies have suggested that surgical tissue injury, postoperative edema, impaired lymphatic drainage, and inadequate closure of surgical layers all contribute to postoperative fluid accumulation.^[5–7]^ Persistent fluid collection may aggravate the local inflammatory response and create conditions favorable for bacterial colonization, thereby increasing the risk of wound-related complications and delaying postoperative recovery.^[8,9]^ Consequently, effective measures to enhance closure integrity and reduce postoperative fluid leakage are of particular clinical interest in posterior fossa surgery.

Tissue sealing membranes are biocompatible materials designed to reinforce tissue closure and reduce postoperative fluid leakage by providing a physical barrier at the surgical site.^[10]^ Such materials have been applied in various surgical specialties and have demonstrated potential benefits in reducing postoperative exudation and promoting wound healing. However, data regarding their application in posterior fossa surgery remain limited, and their effectiveness in preventing postoperative subcutaneous fluid collection in this specific anatomical region has not been fully established.^[11–13]^

Therefore, the present study aimed to evaluate the clinical effectiveness of tissue sealing membranes in posterior fossa surgery, with a particular focus on their role in reducing postoperative subcutaneous fluid collection. In addition, hospitalization duration and surgical costs were analyzed to assess the potential clinical and economic impact of this intervention.

## 2. Materials and methods

### 2.1. Object of study

This retrospective cohort study analyzed adult patients who underwent posterior fossa craniotomy at the Department of Neurosurgery, Sun Yat-Sen Memorial Hospital, Sun Yat-Sen University, between January 2023 and January 2025. Patients aged 18 to 75 years who had a definite indication for posterior fossa craniotomy based on preoperative imaging findings were eligible. Patients with severe dysfunction of major organs such as the heart, lung, or kidney that could compromise surgical tolerance, as well as those with severe coagulation disorders or receiving anticoagulant therapy, were excluded. Additional exclusion criteria were severe cognitive impairment or psychiatric disorders that could interfere with postoperative evaluation, active infections, pregnancy or lactation, known allergy to the sealing membrane material, the presence of other serious conditions affecting postoperative recovery, or a history of previous posterior fossa surgery. The analytic cohort comprised 237 patients with complete 3-month outcome data. The retrospective source records did not permit reliable reconstruction of the total number initially screened before application of the eligibility criteria, and strictly consecutive enrollment could not be verified; this limitation is now explicitly acknowledged and the available patient-selection process is summarized in Figure 2.

**Figure 1.**
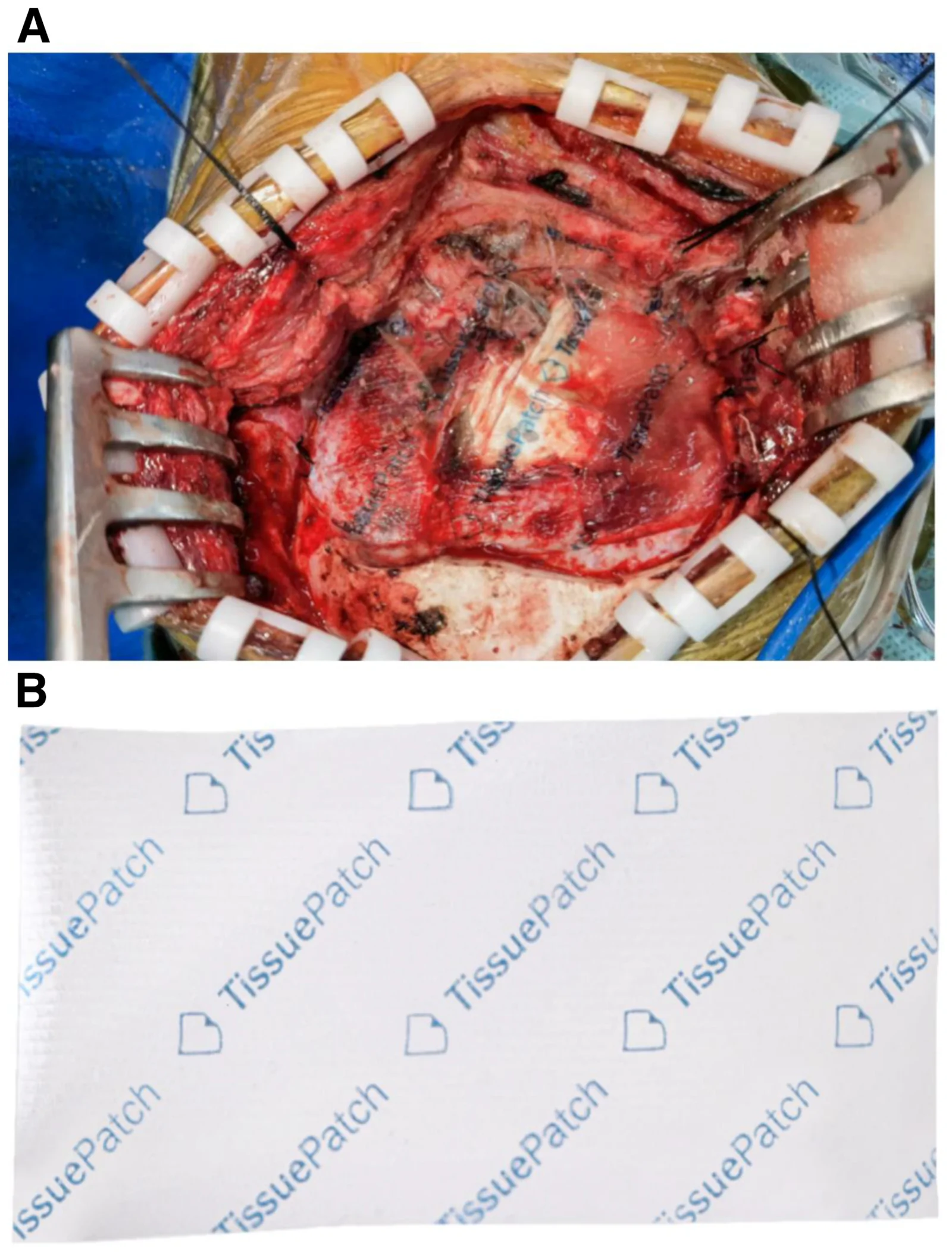
(A) Intraoperative application of an absorbable polymer tissue sealing membrane over the dural suture line. (B) The absorbable polymer tissue sealing membrane used in this study (manufacturer: UK Desumaide Co., LTD.; product name: Sutai).

**Figure 2.**
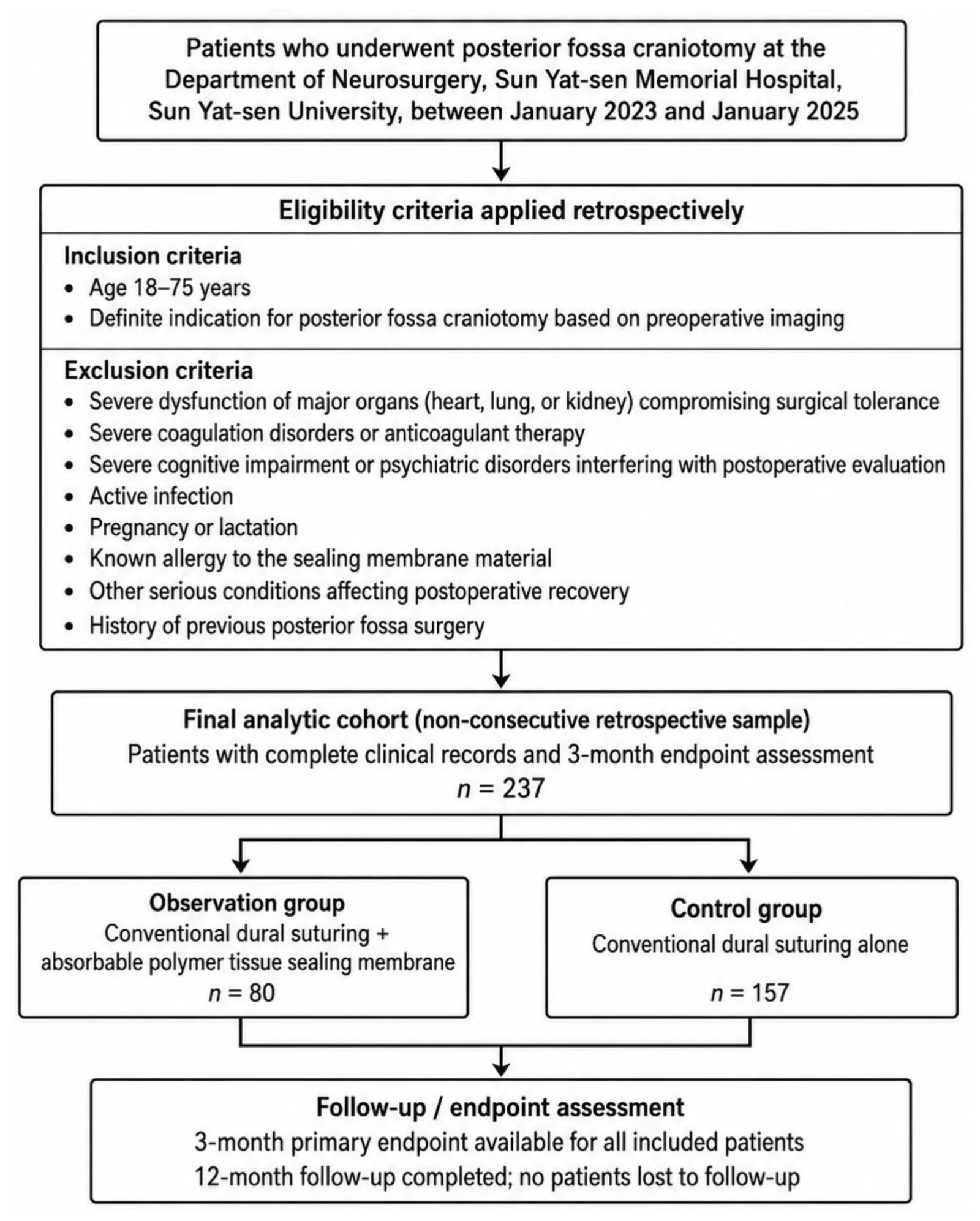
Patient-selection flowchart.

Patients were classified according to whether an absorbable polymer tissue sealing membrane had been applied intraoperatively. The observation group underwent conventional dural suturing combined with application of the sealing membrane, whereas the control group received conventional dural suturing alone. This was not a randomized treatment assignment. Membrane use reflected routine intraoperative practice, and the retrospective dataset did not contain a prospectively defined, standardized indication governing its use. Accordingly, treatment-selection bias and residual confounding related to unrecorded factors influencing the surgeon’s decision cannot be excluded.

This study was reviewed and approved by the Medical Ethics Committee of Sun Yat-sen Memorial Hospital, Sun Yat-sen University (SYSKY-2023-627-01). As this was a retrospective analysis based on anonymized clinical data, the requirement for informed consent was waived by the ethics committee.

### 2.2. Method of repair

In both groups, dural repair was performed by neurosurgeons with more than 8 years of operative experience. When dural defects were encountered intraoperatively, an artificial dural substitute of appropriate size and shape was selected according to the characteristics of the defect and sutured to achieve adequate coverage. After dural closure, a subdural saline injection test was routinely used to assess closure integrity, and additional sutures were placed if cerebrospinal fluid leakage or obvious exudation was detected.

In the Control group, dural repair was carried out using conventional suturing alone. The dura mater was closed in a tension-free fashion with 5 to 0 Prolene sutures, with a suture spacing of approximately 2 to 3 mm. The adequacy of the repair was verified intraoperatively by the saline injection test, and reinforcement sutures were added when necessary until satisfactory tightness was achieved.

In the Observation group, dural suturing was performed using the same technique as that applied in the Control group, followed by placement of an absorbable polymer tissue-sealing membrane (UK Desumaide Co., LTD.; product name: Sutai). A membrane of appropriate size was applied to cover the dural suture line and areas considered mechanically weak (Fig. 1). The membrane was gently pressed onto the dural surface with saline-soaked gauze for approximately 60 seconds to promote adherence. To reduce inter-operator variability in the application technique, all procedures followed the same operative steps described above, and surgeons were familiar with the membrane application procedure.

### 2.3. Observational index

#### 2.3.1. Primary outcome

The primary outcome was postoperative subcutaneous fluid collection within 3 months after surgery. For this retrospective analysis, a case was defined as a clinically apparent fluid collection at or around the incision site (e.g., swelling with increased tension and fluctuation) that was documented by the treating team and, when clinically indicated, evaluated by diagnostic aspiration/puncture. The appearance of aspirated fluid was recorded when available; however, clear fluid alone was not considered sufficient to establish a definite cerebrospinal fluid (CSF) origin. No uniform biochemical assay (e.g., β2-transferrin/β-trace protein) or mandatory imaging criterion was prespecified in the retrospective study protocol for all cases. Accordingly, the study endpoint is reported as postoperative subcutaneous fluid collection rather than confirmed CSF leakage or pseudomeningocele.

#### 2.3.2. Assessment timepoints

During hospitalization, the incision site was assessed at least once daily. After discharge, patients were reviewed in the outpatient clinic at 2 weeks, 1 month, and 3 months after surgery, with additional visits arranged when wound swelling or related symptoms developed. The 3-month assessment (or the latest documented assessment within 3 months) was used for endpoint determination. Imaging or biochemical testing, when obtained for clinical reasons, was considered supportive information but was not required by a standardized study-wide diagnostic algorithm.

#### 2.3.3. Secondary outcomes

Secondary outcomes were hospitalization duration (days from surgery to discharge), total surgical costs (procedure-related in-hospital costs during the index admission), and epithelialization time (days from surgery to complete incision epithelialization recorded in the medical chart).

#### 2.3.4. Follow-up and completeness

Follow-up was conducted through scheduled outpatient visits. When an outpatient visit could not be completed, follow-up was performed by telephone, supplemented by a review of available medical records. Patients were followed for 12 months after surgery for late complications and recurrence. Follow-up for both the 3-month endpoint and the 12-month period was complete, and no patients were lost to follow-up.

#### 2.3.5. Risk-factor analysis

Patients were classified as having postoperative subcutaneous fluid collection or no postoperative subcutaneous fluid collection based on the above definition within 3 months. Candidate variables were evaluated in univariable analyses and then entered into a multivariable logistic regression model to identify factors independently associated with the outcome.

### 2.4. Statistical method

Statistical analyses were conducted using IBM SPSS Statistics version 25.0 (IBM Corp., Armonk). Continuous variables were assessed for normality and are presented as mean ± standard deviation or median (interquartile range), as appropriate. Categorical variables are reported as numbers and percentages. Between-group comparisons (Control group vs Observation group) were performed using the independent-samples *t* test or the Mann–Whitney *U* test for continuous variables, and the chi-square test or Fisher’s exact test for categorical variables. Factors associated with postoperative subcutaneous fluid collection within 3 months were evaluated using logistic regression analysis. Variables of clinical relevance and those showing association in univariable analysis were entered into a multivariable logistic regression model, with results expressed as odds ratios (ORs) and 95% confidence intervals (CIs). Multicollinearity was assessed using variance inflation factors. Hospitalization duration and total surgical costs were analyzed as continuous outcomes, and regression analyses were applied when appropriate. All statistical tests were two-sided, and *P* < .05 was considered statistically significant.

## 3. Results

### 3.1. Baseline characteristics

A total of 237 patients were included in the analytic cohort, including 80 in the Observation group and 157 in the Control group. The mean age was 48.74 ± 17.33 years in the Observation group and 49.29 ± 19.01 years in the Control group (*P* = .829). There were no significant differences between groups in age, sex, disease type, or incision type (all *P* > .05) (Table 1). Figure 2 summarizes the available patient-selection and follow-up pathway.

**Table 1:** The comparison of baseline data for patients included between the 2 groups.

Group	Observation group (n = 80)	Control group (n = 157)	*t/X^2^*	*P*
Age	48.74 ± 17.33	49.29 ± 19.01	0.216	.829
Gender				
Male	38 (47.5%)	65 (41.4%)	0.802	.370
Female	42 (52.5%)	92 (58.6%)		
Disease				
Meningeoma	8 (10.0%)	28 (17.8%)	4.938	.423
Glioma	11 (13.8%)	19 (12.1%)		
Metastatic tumor	8 (10.0%)	8 (5.1%)		
Schwannoma	21 (26.3%)	34 (21.7%)		
Nerve vascular compression syndrome	27 (33.8%)	55 (35.0%)		
Others	5 (6.3%)	13 (8.3%)		
Incision type				
Middle	20 (25.0%)	49 (31.2%)	1.014	.602
Paramedian	3 (3.8%)	6 (3.8%)		
After the sigmoid sinus	57 (71.2%)	102 (65.0%)		

### 3.2. Postoperative subcutaneous fluid collection

Within 3 months after surgery, postoperative subcutaneous fluid collection occurred in 6 patients (7.5%) in the Observation group and 29 patients (18.5%) in the Control group (*P* = .024). Among patients with a documented collection, 21 underwent aspiration, and 14 received additional compression or drainage. Because the endpoint was not uniformly verified by a standardized biochemical or imaging criterion, these events are not presented as confirmed CSF leaks or pseudomeningoceles. No recurrence of the documented subcutaneous fluid collection was recorded during the 12-month follow-up (Table 2).

**Table 2:** Postoperative subcutaneous effusion.

Group	Total Patients(n)	Effusion Cases, n (%)	Local aspiration	Drainage required	*P*-value
Observation	80	6 (7.5%)	4	2	.024
Control	157	29 (18.5%)	17	12	

Subcutaneous effusion was defined as clinically evident fluid collection (swelling, tension, fluctuation) confirmed by aspiration of clear fluid consistent with CSF within 3 months postoperatively.

### 3.3. Risk factor analysis

Logistic regression was performed to identify variables associated with postoperative subcutaneous fluid collection. Univariate analysis showed associations with age, incision type, and sealing membrane use. In multivariable analysis, sealing membrane use was associated with lower odds of postoperative subcutaneous fluid collection (OR = 0.35, 95% CI: 0.14–0.88, *P* = .026), while older age (OR = 1.02 per year, 95% CI: 1.00–1.04, *P* = .045) and paramedian incision (OR = 2.11, 95% CI: 1.02–4.36, *P* = .043) were associated with higher odds (Table 3).

**Table 3:** Logistic regression analysis of risk factors for subcutaneous effusion.

Variable	Univariate OR (95% CI)	*P*-value	Multivariate OR (95% CI)	*P*-value
Age (per yr)	1.02 (1.00–1.04)	.038	1.02 (1.00–1.04)	.045
Gender (male vs female)	1.12 (0.58–2.15)	.742	–	–
Incision: paramedian	2.25 (1.15–4.43)	.018	2.11 (1.02–4.36)	.043
Incision: post-sigmoid	1.47 (0.62–3.45)	.383	–	–
Sealing membrane used	0.35 (0.14–0.88)	.025	0.35 (0.14–0.88)	.026

Reference group for incision type is midline incision.

### 3.4. Postoperative recovery

Mean hospitalization duration was shorter in the Observation group than in the Control group (8.2 ± 2.1 vs 10.5 ± 3.4 days, *P* = .012). No significant difference was found in surgical costs between the 2 groups (¥52,000 ± 13,000 vs ¥50,000 ± 15,000, *P* = .157). Epithelialization time was comparable (12.3 ± 2.5 vs 12.8 ± 3.1 days, *P* = .214) (Table 4).

**Table 4:** Postoperative recovery indicators.

Outcome	Observation goup (n = 80)	Control group (n = 157)	*P*-value
Hospitalization duration (d)	8.2 ± 2.1	10.5 ± 3.4	.012*
Total surgical costs (¥)	52,000 ± 13,000	50,000 ± 15,000	.157
Epithelialization time (d)	12.3 ± 2.5	12.8 ± 3.1	.214

Data are presented as mean ± standard deviation.

* *P* < .05.

## 4. Discussion

This study evaluated the adjunctive use of an absorbable polymer tissue sealing membrane during dural closure in posterior fossa surgery, focusing on postoperative subcutaneous fluid collection. A lower incidence of this outcome was observed in the observation group than in the control group. Prior studies have reported benefits of sealing materials for CSF-related wound complications after cranial procedures.^[14–17]^ However, because the present retrospective endpoint was not uniformly confirmed as CSF by a standardized biochemical or imaging criterion, our results should be interpreted specifically as an association with clinically documented postoperative subcutaneous fluid collection rather than as direct evidence of reduced confirmed CSF leakage or pseudomeningocele.

The absorbable polymer sealing film is engineered as a multilayer construct composed of polymers with complementary functions, including adhesive, hydrophobic, linking, sustained-release, and leakage-prevention layers.^[18]^ The adhesive layer allows rapid attachment to the dural surface, which may enhance early stability along the suture line and reduce the risk of membrane displacement during the initial postoperative period. The hydrophobic layer provides barrier properties against fluid permeation while maintaining mechanical strength, and the linking layer increases tear resistance and overall durability, which may be important when dural tissue is fragile or closure is under tension. The sustained-release layer may contribute to a local milieu supportive of tissue repair, while the leakage-prevention layer can function as an outer seal that limits seepage and reduces blood oozing, and also provides an isolating surface to reduce unwanted adhesion.^[19]^ Clinically, these properties address a practical issue after posterior fossa surgery: even when dural suturing appears satisfactory, microgaps along the suture line or mechanically weak regions may permit low-volume but persistent CSF seepage that can accumulate within the subcutaneous space.

This rationale is supported by prior clinical reports. Ferroli P et al found that 9.2% of high-risk patients developed CSF leakage when tissue sealing membranes were used.^[20]^ Della Puppa et al also reported that sealing membranes were safe and effective in preventing postoperative CSF leakage.^[21]^ These prior studies specifically evaluated CSF leakage, whereas the present study evaluated postoperative subcutaneous fluid collection without uniform biochemical or imaging confirmation of CSF origin. The findings are, therefore, clinically related but should not be treated as identical endpoints. A plausible mechanism is that reinforcement of the dural closure may reduce low-volume fluid seepage into the subcutaneous space, but this mechanism requires prospective confirmation.

Reducing postoperative subcutaneous fluid collection has implications beyond preventing a single postoperative finding. Such collections may increase pain, delay incision healing, require repeated aspiration or drainage, and increase patient anxiety and overall treatment burden. Persistent collections may also predispose to secondary wound complications.^[22,23]^ From a perioperative management standpoint, a lower collection rate may reduce the need for repeated wound interventions and extended monitoring, which could facilitate earlier discharge. Nevertheless, the present study was not designed to determine the biochemical origin of each collection or to establish that each event represented CSF leakage.

The role of sealing membranes in routine practice also warrants consideration. Posterior fossa surgery involves diverse diseases and operative approaches, and the risk of postoperative subcutaneous fluid collection is not uniform. Patient and operative factors: such as age, incision type, dural quality, closure tension, and intraoperative CSF conditions: may influence whether a postoperative collection develops. Importantly, membrane use in this retrospective cohort was not randomized and was not governed by a prospectively documented standardized indication. Therefore, unmeasured factors affecting the intraoperative decision to use the membrane may have differed between groups despite similar measured baseline characteristics and multivariable adjustment.

Several limitations should be acknowledged. First, the retrospective single-center design introduces potential selection bias and limits generalizability. Membrane use was not randomized, and the retrospective dataset did not document a standardized indication for its use; therefore, confounding by indication and other unmeasured treatment-selection factors cannot be excluded. Second, the primary endpoint was a clinically documented postoperative subcutaneous fluid collection. Although aspiration findings were available in some patients, clear fluid alone cannot reliably distinguish CSF from seroma, and a uniform biochemical or imaging confirmation algorithm was not prespecified. Accordingly, the present data should not be interpreted as measuring confirmed CSF leakage or pseudomeningocele. Third, the source records did not permit reliable reconstruction of the number of patients initially screened before application of eligibility criteria, and strictly consecutive enrollment could not be verified. Fourth, although statistical adjustment can mitigate confounding, residual confounding remains possible, especially for factors that are difficult to capture retrospectively, such as subtle differences in closure quality, intraoperative CSF pressure conditions, or surgeon-specific technique. Finally, the sample size may be underpowered for less frequent complications and detailed subgroup analyses, and follow-up beyond 12 months was not available.

Further studies are warranted, including prospective multicenter randomized controlled trials with standardized dural closure protocols, prespecified criteria for membrane use, and standardized diagnostic criteria for postoperative fluid collections. Future studies should ideally distinguish seroma from CSF-related collections using predefined imaging and/or biochemical confirmation when clinically feasible. Longer follow-up would better define safety and durability, particularly among patients at higher risk of CSF-related complications. Mechanistic studies examining dural healing, microleakage at the suture interface, and interactions between membrane properties and tissue repair may help clarify how these membranes exert their effects and refine patient-selection and application strategies.

## 5. Conclusion

In this single-center retrospective cohort of posterior fossa surgery, patients who received an absorbable polymer tissue sealing membrane in addition to standard dural suturing had a lower 3-month rate of postoperative subcutaneous fluid collection and a shorter length of hospital stay, while procedure-related costs were comparable between groups. These findings support further prospective evaluation of the membrane as an adjunct to dural closure but do not establish causality. Because exposure assignment was non-randomized and the postoperative collections were not uniformly confirmed as CSF by standardized biochemical or imaging criteria, larger prospective multicenter studies with standardized patient-selection, outcome definitions, and longer follow-up are needed.

## Acknowledgments

The authors express their gratitude to the dedicated medical and nonmedical staff for their tireless efforts and contributions.

## Author contributions

**Conceptualization:** Weilong Chen, Zhenghao Liu, Xin Mei, Jina Chen, Yuefei Deng.

**Data curation:** Weilong Chen, Zhenghao Liu, Xin Mei, Jina Chen, Yuefei Deng.

**Formal analysis:** Weilong Chen, Zhenghao Liu, Xin Mei, Jina Chen, Yuefei Deng.

**Funding acquisition:** Yuefei Deng.

**Investigation:** Yuefei Deng.

**Writing – original draft:** Yuefei Deng.

**Writing – review & editing:** Yuefei Deng.

## References

[1] Turkistani AN Alsharif TH Aldhafeeri WF Aljohani S Alomar S. Medical management for cerebellar mutism syndrome following posterior fossa surgery: a systematic review. Clin Neurol Neurosurg. 2024;242:108352. 10.1016/j.clineuro.2024.108352 38823197

[2] Bopp MHA Grote A Gjorgjevski M Pojskic M Saß B Nimsky C. Enabling navigation and augmented reality in the sitting position in posterior fossa surgery using intraoperative ultrasound. Cancers (Basel). 2024;16:1985. 10.3390/cancers16111985 PMC11171013 38893106

[3] Kameda-Smith MM Ragulojan M Elliott C; McMaster Pediatric Brain Tumour Study Group (PBTSG) and the Canadian Neurosurgery Research Collaborative (CNRC). McMaster Pediatric Brain Tumour Study Group (PBTSG) and the Canadian Neurosurgery Research Collaborative (CNRC). National multicentered retrospective review of clinical and intraoperative factors associated with the development of cerebellar mutism after pediatric posterior fossa tumor resection. Childs Nerv Syst. 2024;40:1339–47. 10.1007/s00381-024-06292-0 38279985

[4] Varengue R Delion M De Carli E. Evaluation of safety of fluoxetine for cerebellar mutism syndrome in children after posterior fossa surgery. Arch Pediatr. 2024;31:231–7. 10.1016/j.arcped.2023.10.010 38485568

[5] Chen A Zhou R Yao X Ai M Sun T. Neuroendoscopic treatment of giant cystic craniopharyngioma in the foramen magnum: report of two cases. Childs Nerv Syst. 2021;37:2387–90. 10.1007/s00381-020-04965-0 33169209

[6] Orlev A Jackson CM Luksik A. Natural history of untreated transverse/sigmoid sinus thrombosis following posterior fossa surgery: case series and literature review. Operative Neurosurg (Hagerstown, Md.). 2020;19:109–16. 10.1093/ons/opz396 31853546

[7] Ahmadian N van Baarsen KM Robe PAJT Hoving EW. Association between cerebral perfusion and paediatric postoperative cerebellar mutism syndrome after posterior fossa surgery-a systematic review. Childs Nerv Syst. 2021;37:2743–51. 10.1007/s00381-021-05225-5 PMC8423702 34155533

[8] Dilmen OK Akcil EF Vehid H Tunali Y. Cerebral oxygenation assessed by near-infrared spectroscopy in the sitting and prone positions during posterior fossa surgery: a prospective, randomized clinical study. Braz J Anesthesiol. 2023;73:589–94. 10.1016/j.bjane.2021.09.016 PMC10533958 34626757

[9] Lewis D Sethuraman C Varthalitis D. Rapid ossification of a giant post-operative occipital pseudomeningocele following posterior fossa surgery. Childs Nerv Syst. 2023;39:1347–51. 10.1007/s00381-023-05829-z PMC10167119 36680566

[10] Wu KC Freedman BR Kwon PS. A tough bioadhesive hydrogel supports sutureless sealing of the dural membrane in porcine and ex vivo human tissue. Sci Transl Med. 2024;16:eadj0616. 10.1126/scitranslmed.adj0616 PMC11145396 38507468

[11] Rossi N Bejar-Chapa M Giorgino R. Photosealed neurorrhaphy using autologous tissue. Int J Mol Sci. 2024;25:6958. 10.3390/ijms25136958 PMC11241074 39000065

[12] Badaut J Ghersi-Egea JF Thorne RG Konsman JP. Blood-brain borders: a proposal to address limitations of historical blood-brain barrier terminology. Fluids Barriers CNS. 2024;21:3. 10.1186/s12987-023-00478-5 PMC10770911 38183042

[13] Štefl M Takamiya M Middel V. Caveolae disassemble upon membrane lesioning and foster cell survival. iScience. 2024;27:108849. 10.1016/j.isci.2024.108849 PMC10831942 38303730

[14] Alam JM Maruyama T Noshiro D. Complete set of the Atg8-E1-E2-E3 conjugation machinery forms an interaction web that mediates membrane shaping. Nat Struct Mol Biol. 2024;31:170–8. 10.1038/s41594-023-01132-2 38057553

[15] Zhou F Zhang Y Gao J Xiang G Li Z Cai L. Reconstruction of foot and ankle defects using the vaccum sealing drainage versus the induced-membrane the elderly: a retrospective comparative study. Int Wound J. 2024;21:e14362. 10.1111/iwj.14362 PMC10781590 37605359

[16] Saleh MHA Dias DR Ravida A Wang HL. Root surface biomodification in periodontal therapy: biological rationale and clinical applications. Periodontol 2000. 2024:12576. 10.1111/prd.12576 38978341

[17] Fang Y Zhang Y Bi S. Securing LYTAC with logic-identification system for cancer cell-selective membrane protein degradation. Small. 2024;20:e2310039. 10.1002/smll.202310039 38431928

[18] Yang L Ou Z Jiang G. Research progress of elastomer materials and application of elastomers in drilling fluid. Polymers (Basel). 2023;15:918. 10.3390/polym15040918 PMC9959665 36850203

[19] Kurochkin MA Sindeeva OA Abdurashitov AS Pyataev NA Gorin DA Sukhorukov GB. In vivo laser-induced vasoactive microenvironmental setting via a stimuli-responsive microstructured depot. Biomacromolecules. 2023;24:3051–60. 10.1021/acs.biomac.3c00125 37289998

[20] Ferroli P Acerbi F Broggi M. A novel impermeable adhesive membrane to reinforce dural closure: a preliminary retrospective study on 119 consecutive high-risk patients. World Neurosurg. 2013;79:551–7. 10.1016/j.wneu.2011.09.022 22120260

[21] Della Puppa A Rossetto M Scienza R. Use of a new absorbable sealing film for preventing postoperative cerebrospinal fluid leaks: remarks on a new approach. Br J Neurosurg. 2010;24:609–11. 10.3109/02688697.2010.500413 20868249

[22] Roman A Tufegdzic B Lamperti M Pinto V Roser F. Before the knife: a detailed step-by-step description of an optimized semi-sitting position in posterior fossa surgery. World Neurosurg. 2023;172:e241–9. 10.1016/j.wneu.2022.12.137 36608791

[23] Karn M Mahato BK Sah R Kandel D Sapkota S. “You Sleep, You Die”: a rare clinical case of ondine’s curse after posterior fossa surgery. Case Rep Surg. 2023;2023:3113428. 10.1155/2023/3113428 PMC10506874 37727799

